# A conformational epitope in placental malaria vaccine antigen VAR2CSA: What does it teach us?

**DOI:** 10.1371/journal.ppat.1011370

**Published:** 2023-05-25

**Authors:** Justin Y. A. Doritchamou, Jonathan P. Renn, Lars Hviid, Patrick E. Duffy

**Affiliations:** 1 Laboratory of Malaria Immunology and Vaccinology, National Institute of Allergy and Infectious Diseases, NIH, Bethesda, Maryland, United States of America; 2 Centre for Medical Parasitology, Department of Microbiology and Immunology, University of Copenhagen and Department of Infectious Diseases, Rigshospitalet, Copenhagen, Denmark; University of Wisconsin Medical School, UNITED STATES

## Abstract

VAR2CSA is the *Plasmodium falciparum* variant surface antigen that mediates binding of infected erythrocytes to chondroitin sulfate A (CSA) and their sequestration in intervillous spaces of the placenta, leading to placental malaria (PM). Relatively high polymorphism in VAR2CSA sequences has hindered development of a vaccine that induces broadly neutralizing immunity. Recent research has highlighted that a broadly reactive human monoclonal antibody, called PAM1.4, binds to multiple conserved residues of different subfragments of VAR2CSA, forming a conformational epitope. In this short perspective, we describe evidence that residues located in the interdomain-1 fragment of VAR2CSA within the PAM1.4 binding epitope might be critical to broad reactivity of the antibody. Future investigation into broadly reactive anti-VAR2CSA antibodies may be important for the following: (1) identification of similar conformation epitopes targeted by broadly neutralizing antibodies; and (2) understanding different immune evasion mechanisms used by placenta-binding parasites through VAR2CSA polymorphism in critical epitopes.

## VAR2CSA and the development of placental malaria vaccine

Pregnant women in malaria-endemic areas are at risk of placental malaria (PM), which is characterized by the accumulation of *Plasmodium falciparum*–infected erythrocytes in the placenta. Placenta-sequestering parasites preferentially express VAR2CSA, a member of the PfEMP1 family of adhesive parasite proteins [1], which selectively binds chondroitin sulfate A (CSA) on the syncytiotrophoblasts in the placenta. Women become resistant to PM over successive pregnancies, and this parity-dependent protection has been linked to the acquisition of anti-VAR2CSA antibodies, supporting this protein as the lead candidate for a PM vaccine. High protein sequence polymorphism in VAR2CSA [2–4] represents a major challenge in the development of a VAR2CSA-based vaccine that induces broadly neutralizing immunity. The full-length VAR2CSA extracellular region is a large cysteine-rich protein fragment that comprises several Duffy Binding-Like (DBL) domains and interdomain (ID) regions. Two VAR2CSA N-terminal subunits containing the minimal CSA-binding fragment [5] have been tested in Phase I clinical trials [6,7] but did not induce strain-transcending neutralizing immunoglobulin G (IgG). This finding highlights the need for VAR2CSA-based vaccines to display conserved and functionally important antibody epitopes to achieve a broadly neutralizing activity.

Recent studies have described the molecular structure of the full-length VAR2CSA ectodomain, revealing that VAR2CSA is composed of a stable core (NTS-DBL4) with CSA-binding channel(s) and a flexible arm (DBL5-6) [8,9]. Allelic variation in VAR2CSA proteins did not seem to affect this structure [8], suggesting that all or most VAR2CSA variants adopt a similar fold. Hence, an antibody recognizing a conserved structure-based epitope displayed by VAR2CSA full-length ectodomain is likely to exhibit broad reactivity, whereas the existing subunit vaccines were not designed to stimulate the production of antibodies that recognize this type of epitope. These works provide a blueprint to map conserved or critical epitopes on the VAR2CSA surface.

## A broadly reactive monoclonal antibody to VAR2CSA

The human monoclonal antibody PAM1.4 was generated using immortalized B cells obtained from a malaria-exposed pregnant woman [10]. Recently, an in-depth characterization of this human monoclonal antibody revealed that PAM1.4 binds a highly conserved conformational epitope involving residues in the ID1, DBL2, ID2, and DBL4 domains of VAR2CSA [11]. The in silico analysis of sequence conservation (among the amino acid residues involved in this conformational epitope) indicated that residues in DBL2, ID2, and DBL4 fragments of VAR2CSA are relatively conserved, with residues Y958 and R959 in FCR3 DBL2 the least conserved contact residues with PAM1.4. However, ID1 residues were semiconserved with residues K510-R511 (KR) of FCR3 VAR2CSA absent from 24% of the analyzed VAR2CSA variant sequences [11]. Importantly, the VAR2CSA sequences lacking the PAM1.4-binding residues KR in ID1 cluster in a clade of variants that share a distinct type 2 dimorphic sequence motif (DSM) in ID1 and constitute about a quarter of known VAR2CSA sequences [3]. Whether PAM1.4 binds to VAR2CSA variants exhibiting Type 2 ID1 has not been investigated.

## PAM1.4 binds variants of VAR2CSA with variable levels of reactivity

**Fig 1:**
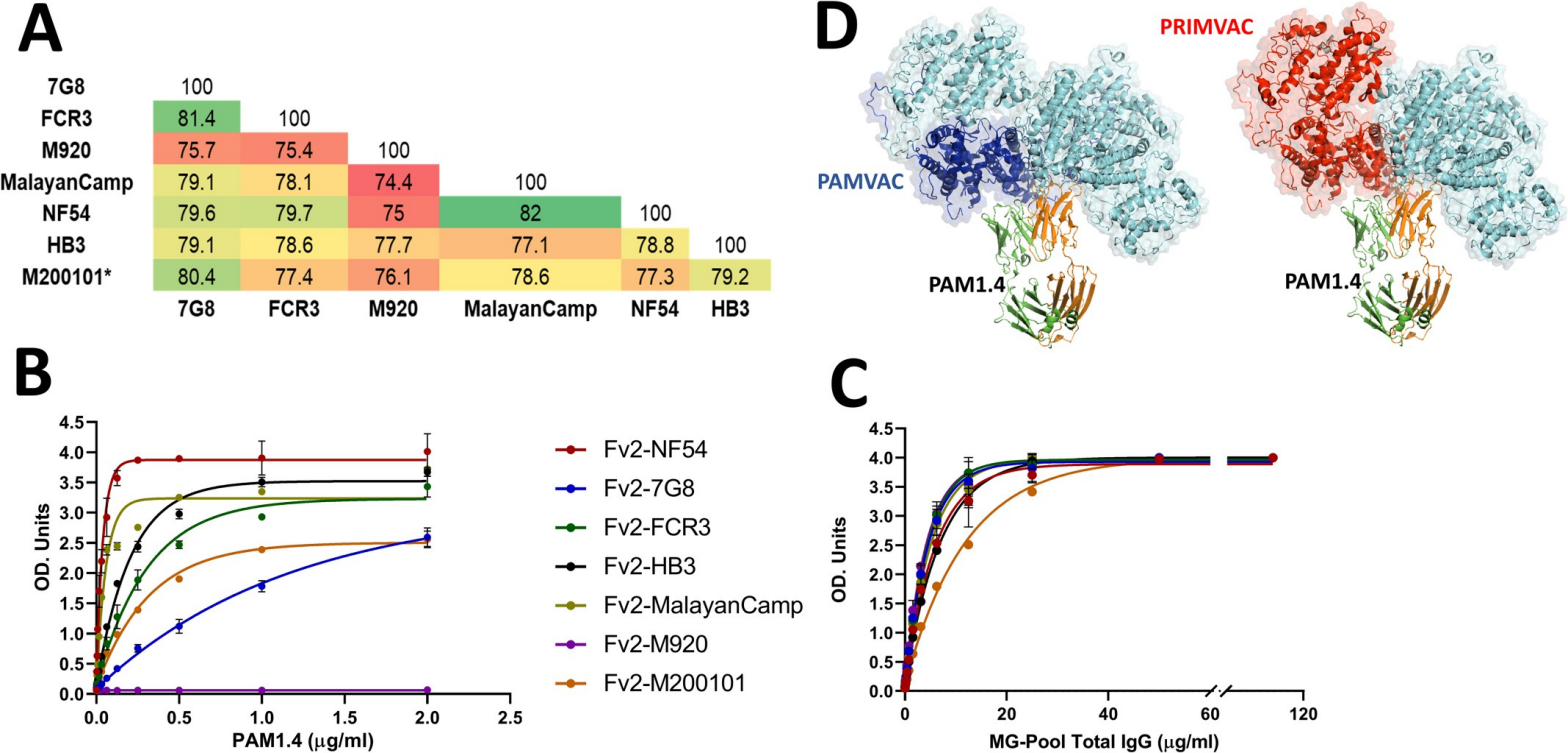
PAM1.4-binding profile to genetically diverse VAR2CSA recombinants. VAR2CSA protein sequence diversity of the 7 full-length VAR2CSA ectodomain has been analysed to highlight **(A)** sequence identity within the 7 phylogenetically distant alleles of VAR2CSA, reported in Renn and colleagues [12], by multiple alignment of NTS-DBL6 fragments (ranged from 2,640 to 2,723 amino acids) using BioEdit version 7.0.5.3 software. **(B)** The structure of VAR2CSA complex with PAM1.4 (PDB:7Z12) reported in Raghavan and colleagues [11] was used to map the PAMVAC (in blue) and PRIMVAC (in red) vaccine boundaries, while VH:VL chains of PAM1:4 are highlighted in orange and green. **(C)** The binding level of human monoclonal IgG PAM1.4 and **(D)** pooled purified IgG from MG to the 7 full-length VAR2CSA (Fv2) recombinants was measured by ELISA. OD units are reported. IgG, immunoglobulin G; MG, multigravidae; OD, optical density.

We have previously expressed and characterized recombinants of 7 genetically diverse full-length VAR2CSA ectodomains (representing major clusters of *var2csa* sequences) (**Fig 1A**), in a mammalian expression system [12]. Among the expressed VAR2CSA variants, M200101 has an additional DBL7ɛ domain downstream of DBL6ɛ [13], and the M920 (harboring the Type 2 DSM in ID1) was the least similar to the other 6 variants of full-length VAR2CSA [12].

Using these VAR2CSA proteins, we profiled human monoclonal antibody PAM1.4 binding to VAR2CSA variants, to investigate the importance of PAM1.4-binding residues on VAR2CSA with regard to the broad reactivity of the antibody. Variable levels of PAM1.4 reactivity to several variants of VAR2CSA were observed, with the highest binding measured against NF54 variant and the lowest seen with 7G8 variant (**Fig 1B**). Notably, PAM1.4 did not bind M920, suggesting that sequence variation may affect VAR2CSA fold or modifies posttranslational modification of the protein, thereby affecting the display of conformational epitope. However, polyclonal IgG pooled from multigravidae bound to all 7 VAR2CSA variants, with the lowest level of binding observed for M200101 variant, which has an atypical structure with an extra DBL domain downstream of DBL6ɛ (**Fig 1C**). These observations highlight the potential of inducing antibodies that target multiple protective epitopes in VAR2CSA, to develop an effective PM vaccine.

## Importance of strain-transcending functional antibody in PM vaccine development

Sequence polymorphism in VAR2CSA represents an important issue for variant selection in PM vaccine development, as many studies of *var2csa* genetic diversity have highlighted significant sequence variation across the gene [2,3,14]. Acquisition of protective immunity to *P*. *falciparum* malaria in general is often speculated to result from the acquisition and accumulation of a broad repertoire of PfEMP1 variant-specific antibodies over successive exposure to the parasites [15,16]. However, the rapid acquisition of antibodies with CSA-binding inhibition activity in paucigravid women [17,18] supports a model where protective antibodies targeting conserved epitopes in VAR2CSA are acquired already at the first episode of PM. The work by Raghavan and colleagues [11] provides evidence that they include discontinuous epitopes only displayed by the full-length VAR2CSA ectodomain. Hence, the identification of broadly cross-reactive epitopes in VAR2CSA, like that of PAM1.4, would critically inform VAR2CSA-based PM vaccine development. Although some variants might escape antibodies targeting conformational epitopes in VAR2CSA, key antibody-binding residues shared by divergent VAR2CSA haplotypes might offer the possibility to combine a limited number of antigenic components in a PM vaccine cocktail.

Broadly neutralizing monoclonal antibodies binding to conserved VAR2CSA epitopes are likely to be common among women naturally exposed to PM parasites, as a single full-length VAR2CSA ectodomain can purify broadly neutralizing activity in antibodies from PM-resistant multigravid women [19]. In contrast, similar experiments with VAR2CSA subunits covering ID1 to DBL5 domains from different alleles failed to purify or deplete broadly neutralizing activity from multigravida IgG [20]. Thus, naturally acquired broadly neutralizing antibodies could be targeting conformational epitopes on VAR2CSA.

## Conclusions and future considerations

Sequence variation in VAR2CSA likely impacts epitope availability and, thus, antibody binding. Much remains to be learned about the molecular details of VAR2CSA including the epitopes capable of inducing broadly neutralizing immunity in PM-exposed pregnant women. As yet, no VAR2CSA antibody that binds to a fully conserved VAR2CSA epitope has been identified. Such epitopes, especially those targeted by neutralizing antibodies, would significantly advance the development of a potent VAR2CSA-based vaccine. Antibody to PAM1.4-like strain-transcending epitopes may fail to be induced by VAR2CSA subunit immunogens (**Fig 1D**), as seen in humans immunized with VAR2CSA fragments who mounted limited serum functional activity against heterologous strains [6,7]. Paths to identify broadly neutralizing epitopes for PM vaccine may include the use of full-length VAR2CSA ectodomain to differentially map epitopes of naturally acquired antibodies by PM-resistant multigravid women over those of PM-exposed primigravidae.

Due to the limited success of the malaria vaccine candidates, notably those targeting blood stage antigens, the study of conformational epitopes displays by vaccine antigen or complex of proteins will boost the ongoing effort to optimize these vaccines. Such observation was previously demonstrated in AMA1-RON2L complex report to indicate that improved efficacy of the complex vaccine over AMA1 alone can be obtained when AMA1 is coupled to a rhoptry neck protein 2 (RON2) peptide to improve the antibody neutralizing activity [21]. Broadly neutralizing human monoclonal antibody binding to conformational epitopes has been also described in other infectious disease pathogens such as respiratory syncytial virus (RSV) [22]. Overall, identifying conformational epitopes composed of highly conserved residues and targeted by broadly neutralizing antibody will enhance the development of efficacious vaccines against several infectious disease pathogens, including malaria.
