# Correction: A conformational epitope in placental malaria vaccine antigen VAR2CSA: What does it teach us?

**DOI:** 10.1371/journal.ppat.1011503

**Published:** 2024-01-29

**Authors:** Justin Y. A. Doritchamou, Jonathan P. Renn, Lars Hviid, Patrick E. Duffy

In Fig 1, panels B, C and D are mislabeled as D, B and C respectively. The authors have provided a corrected version here.

**Fig 1:**
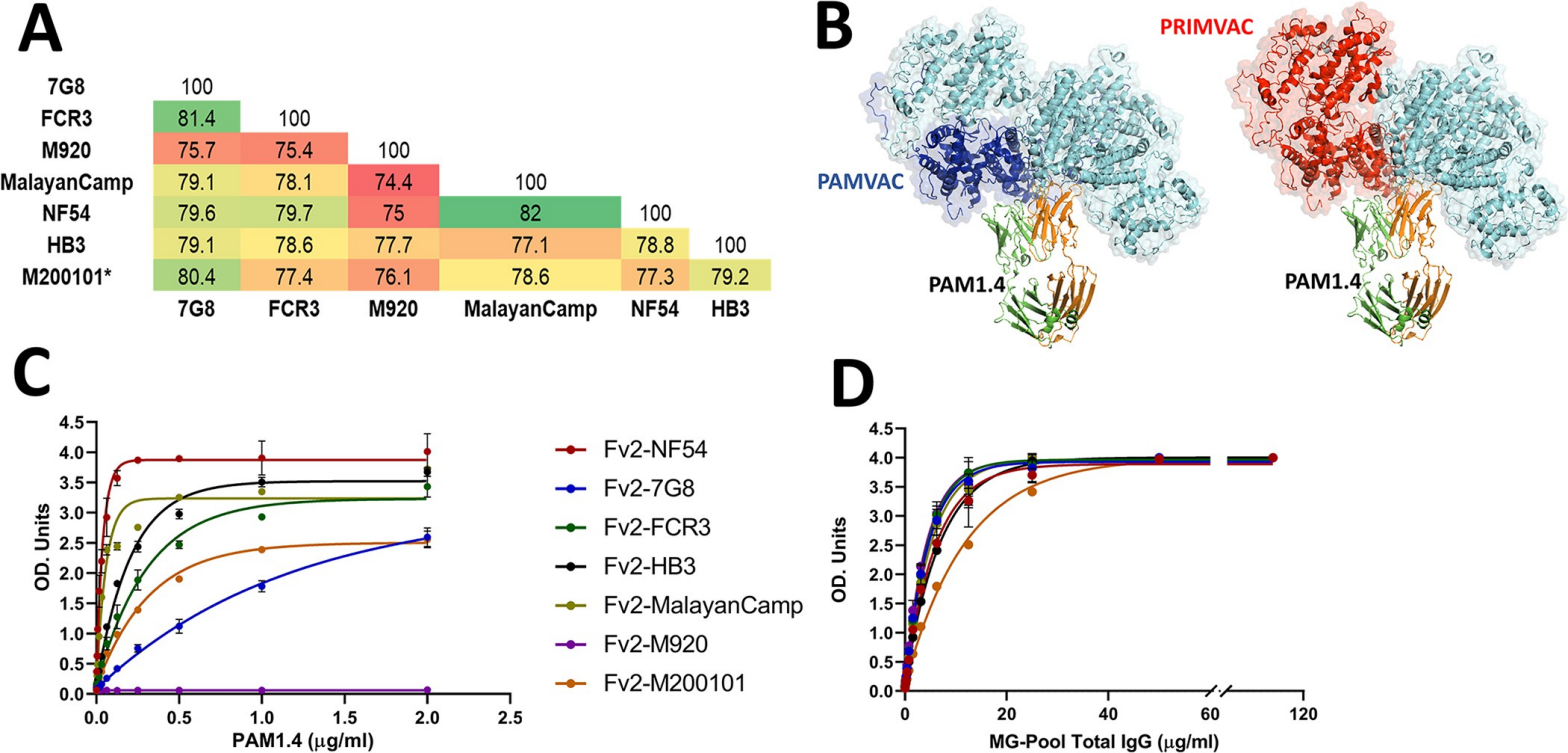
PAM1.4 binding profile to genetically diverse VAR2CSA recombinants. VAR2CSA protein sequence diversity of the seven full-length VAR2CSA ectodomain has been analysed to highlight (A) sequence identity within the seven phylogenetically distant alleles of VAR2CSA, reported in Renn et al. [12], by multiple alignment of NTS-DBL6 fragments (ranged from 2640 to 2723 amino acids) using BioEdit version 7.0.5.3 software. (B) The structure of VAR2CSA complex with PAM1.4 (PDB:7Z12) reported in Raghavan et al. [11] was used to map the PAMVAC (in blue) and PRIMVAC (in red) vaccine boundaries, while VH:VL chains of PAM1:4 are highlighted in orange and green. (C) The binding level of human monoclonal IgG PAM1.4 and (D) pooled purified IgG from multigravidae (MG) to the seven full-length VAR2CSA (Fv2) recombinants was measured by ELISA. Optical density (OD) units are reported.
